# Characterisation and comparative genomics of three new *Varanus*-associated *Borrelia* spp. from Indonesia and Australia

**DOI:** 10.1186/s13071-023-05937-4

**Published:** 2023-09-05

**Authors:** Alexander William Gofton, Angel Popa-Baez, Ai Takano, Kari Soennichsen, Michelle Michie, Makenna Short, Supriyono Supriyono, Jack Pascoe, Sue Cusbert, Robert Mulley

**Affiliations:** 1 Health and Biosecurity, Commonwealth Scientific and Industrial Research Organisation (CSIRO), Canberra, Australia; 2 Department of Veterinary Medicine, Joint Faculty of Veterinary Medicine, Yamaguchi University, Yamaguchi, Japan; 3 Institute for Applied Ecology, University of Canberra, Canberra, Australia; 4 Department of Animal Diseases and Veterinary Health, Bogor Agricultural University, Bogor, Indonesia; 5 School of Agricultural and Ecosystem Sciences, University of Melbourne, Melbourne, Australia; 6 School of Science and Health, Western Sydney University, Penrith, Australia

**Keywords:** *Borrelia*, Reptile-associated *Borrelia*, Ticks, Tick-borne disease, Varanidae, Monitor lizard

## Abstract

**Background:**

*Borrelia* are important disease-causing tick- and louse-borne spirochaetes than can infect a wide variety of vertebrates, including humans and reptiles. Reptile-associated (REP) *Borrelia*, once considered a peculiarity, are now recognised as a distinct and important evolutionary lineage, and are increasingly being discovered worldwide in association with novel hosts. Numerous novel *Borrelia* spp. associated with monitor lizards (*Varanus* spp.) have been recently identified throughout the Indo-Pacific region; however, there is a lack of genomic data on these *Borrelia*.

**Methods:**

We used metagenomic techniques to sequence almost complete genomes of novel *Borrelia* spp. from *Varanus varius* and *Varanus giganteus* from Australia, and used long- and short-read technologies to sequence the complete genomes of two strains of a novel *Borrelia* sp. previously isolated from ticks infesting *Varanus salvator* from Indonesia. We investigated intra- and interspecies genomic diversity, including plasmid diversity and relatedness, among *Varanus*-associated *Borrelia* and other available REP *Borrelia* and, based on 712 whole genome orthologues, produced the most complete phylogenetic analysis, to the best of our knowledge, of REP *Borrelia* to date.

**Results:**

The genomic architecture of *Varanus*-associated *Borrelia* spp. is similar to that of *Borrelia* spp. that cause relapsing fever (RF), and includes a highly conserved megaplasmid and numerous smaller linear and circular plasmids that lack structural consistency between species. Analysis of PF32 and PF57/62 plasmid partitioning genes indicated that REP *Borrelia* plasmids fall into at least six distinct plasmid families, some of which are related to previously defined *Borrelia* plasmid families, whereas the others appear to be unique. REP *Borrelia* contain immunogenic variable major proteins that are homologous to those found in *Borrelia* spp. that cause RF, although they are limited in copy number and variability and have low sequence identities to RF variable major proteins. Phylogenetic analyses based on single marker genes and 712 single copy orthologs also definitively demonstrated the monophyly of REP *Borrelia* as a unique lineage.

**Conclusions:**

In this work we present four new genomes from three novel *Borrelia*, and thus double the number of REP *Borrelia* genomes publicly available. The genomic characterisation of these *Borrelia* clearly demonstrates their distinctiveness as species, and we propose the names *Borrelia salvatorii*, ‘*Candidatus* Borrelia undatumii’, and ‘*Candidatus* Borrelia rubricentralis’ for them.

**Graphical Abstract:**

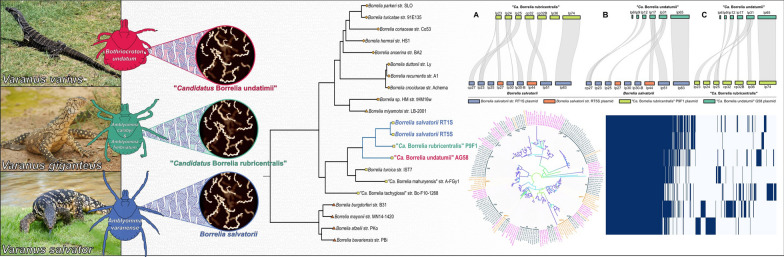

**Supplementary Information:**

The online version contains supplementary material available at 10.1186/s13071-023-05937-4.

## Background

*Borrelia* are globally distributed tick- and louse-borne spirochetes that can infect a wide variety of vertebrate hosts, including humans. *Borrelia* spp. are ecologically diverse and broadly cluster into four distinct evolutionary lineages that reflect their host and vector associations, ecological life cycles, physiology, and human disease manifestations [1]. These lineages include the* Ixodes*-transmitted Lyme borreliosis (LB) clade (*Borreliella*), the Argasidae-transmitted and Ixodidae-transmitted relapsing fever (RF) clades, and the reptile-associated (REP) clade, which is dominated by reptile-infecting species transmitted by Metastriata tick vectors, such as *Borrelia turcica*, but also includes diverse species such as ‘*Candidatus* (*Ca*.) Borrelia tachyglossi’ from Australian echidnas and ‘*Ca*. Borrelia mahuryensis’ found in passerine birds from French Guinea [2, 3].

The RF and LB clades include important human pathogens and have been widely studied for many years. However, REP *Borrelia* were discovered relatively recently and have not received similar scientific attention. Nevertheless, molecular techniques have improved our ability to detect and classify *Borrelia*, and putative REP *Borrelia* are increasingly reported from tortoises, snakes, and *Varanus* (monitor lizards), and even the toad *Rhinella horribilis* [4–11].

In particular, the number of REP *Borrelia* associated with *Varanus* spp. (varanid) hosts has grown rapidly, with novel putative species found in ticks collected from *Varanus exanthematicus* from Tanzania [9], *Varanus varius* from Australia [7], *Varanus salvator* from Indonesia [8], *V*. *salvator* and *Varanus bengalensis* from Thailand [5], and *V. bengalensis* from Pakistan [12]. These *Borrelia* have been found predominantly in association with *Amblyomma* spp. ticks, including *Amblyomma varanense* and *Amblyomma gervaisi*, but also with the tick *Bothriocroton undatum* in Australia, although it has not been established whether these ticks are true *Borrelia* vectors as only blood-fed specimens have been analysed. Phylogenetic analysis of 16S ribosomal RNA (rRNA) and *flaB* sequences indicated that varanid-associated *Borrelia* form a monophyletic clade within the REP *Borrelia* lineage that is distinct from other *Borrelia* clades [8].

*Borrelia* have unique genomes that contain a single linear chromosome and a multitude of both linear and circular plasmids, which can comprise up to 40% of their total genomic content. While the chromosomes of all *Borrelia* species are highly conserved, their plasmids are highly variable, structurally plastic, and contain a high proportion of repetitive genomic elements [13]. In addition, some core plasmids contain critical proteins required for transmission and host invasion, while other accessory plasmids are not critical for *Borrelia* survival but may confer phenotypes that influence their pathogenicity and clinical manifestations [14]. Because of their structural complexity and genetic repetitivity, long-read sequencing technologies are required to properly assemble *Borrelia* plasmids [15].

Genomic data are a critical resource that underpin our understanding of *Borrelia* evolution, population dynamics, host and vector adaptation, and pathogenesis [16]. While a wealth of genomic resources exists for many Argasidae-transmitted RF species and LB species, genomic resources are lacking for most REP species due to their ad hoc discovery and the lack of cultivated isolates. However, the genomes of three REP species, *Borrelia turcica*, ‘*Ca*. Borrelia tachyglossi’, and ‘*Ca*. Borrelia mahuryensis’, have been sequenced [2, 3]. The genome of *B. turcica* is considered complete as it was sequenced from a pure culture with Illumina and PacBio technologies, while the genomes of ‘*Ca*. B. tachyglossi’, and ‘*Ca*. B. mahuryensis’ are thus far incomplete, particularly their plasmid fractions, as they were assembled from Illumina data only directly from an engorged tick and a low passage cultured isolate, respectively [2, 3].

Analysis of these genomes indicates that REP *Borrelia* genomes have a RF-like architecture, including chromosomal synapomorphies, such as the presence of the GlpQ antigen, a single 23S rRNA gene, and a large linear megaplasmid [2, 3]. However, REP genomes also contain distinct genomic features that differentiate them from both the RF and LB species, such as the inclusion of putative maltose metabolism genes *glvA* and *glvC* in the rRNA operon [10], and a highly reduced set of antigenic variable membrane proteins (vmps) compared to their RF counterparts, and numerous genes that are homologous to genes found in LB species that were subsequently lost by all RF species [2, 3]. Interrogating the genomes of REP *Borrelia* spp. is an alternative approach to thorough ecological or epidemiological studies, which have as yet not been performed for any of these species, that can be used to gain insights into their unique biology and evolution and may assist in determining their relevance for human and animal health.

Here, we sequenced four additional REP *Borrelia* genomes from three novel *Borrelia* species from varanids from Indonesia and Australia. Indonesian *Borrelia* were previously isolated from the midgut and salivary glands of engorged *A. varanense* ticks collected from an individual *V. salvator* in Bogor, Indonesia [8]. Australian *Borrelia* were identified during this study by polymerase chain reaction (PCR) on ethanol-preserved *Amblyomma* and *Bothriocroton* ticks from *V. giganteus* and *V. varius*, respectively, and their genomes reconstructed by metagenomic sequencing.

## Methods

### Australian varanid tick samples

**Fig. 1:**
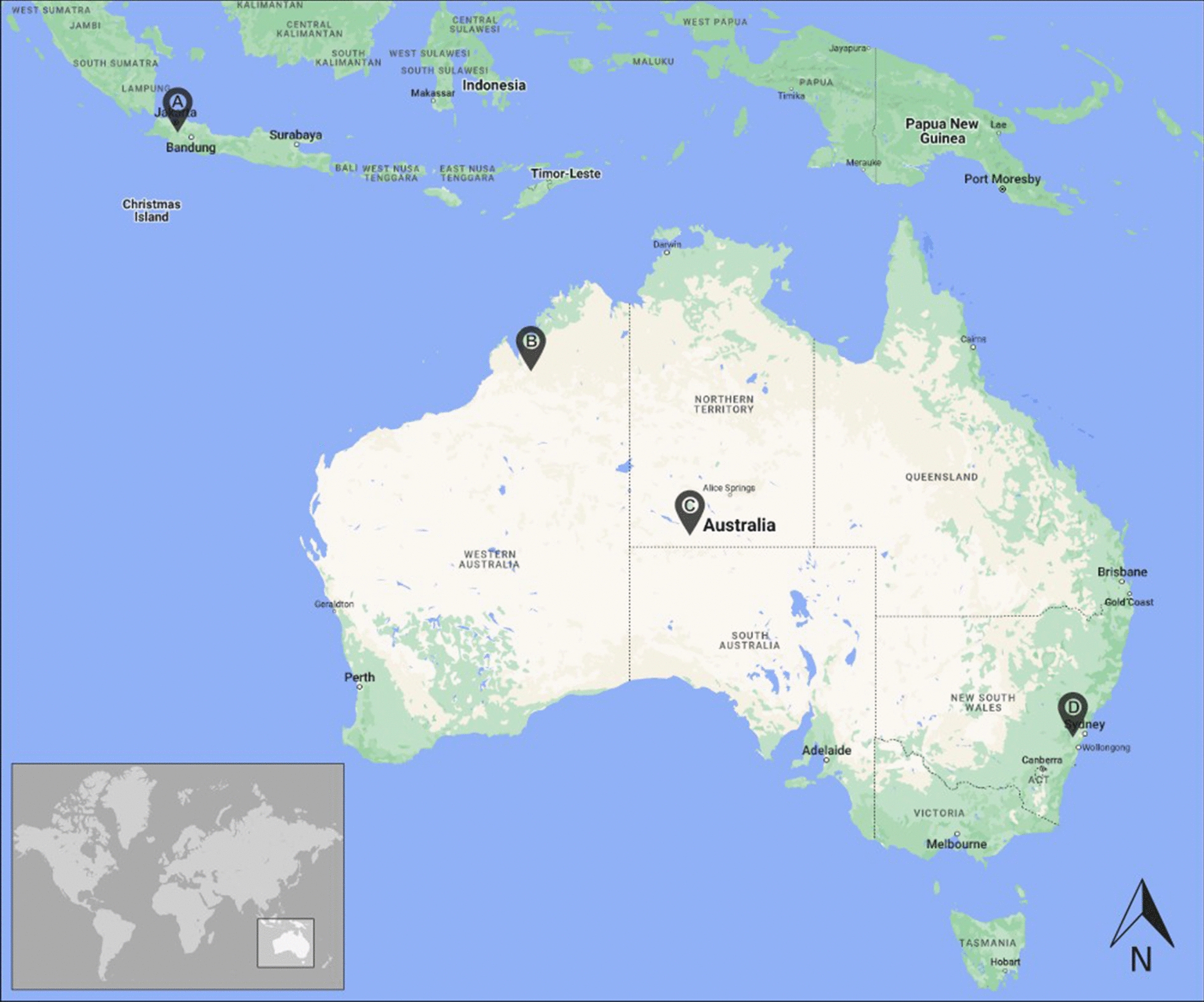
Map of the approximate collection locations of the monitor lizards from which tick cohorts were screened for *Borrelia*. *Varanus salvator* (Bogor, West Java, Indonesia) (*A*); *Varanus acanthurus* (Fitzroy River basin, Western Australia) (*B*); *Varanus giganteus* (Mt. Conner, Northern Territory) (*C*); *Varanus varius* (Burragorang Valley, New South Wales) (*D*)

Three cohorts of Australian varanid ticks were screened for *Borrelia*, comprising a cohort of *Bothriocroton undatum* from *Varanus varius* from the Burragorang Valley, New South Wales [17], a cohort of *Amblyomma calabyi*, *Amblyomma fimbriatum*, and *Amblyomma limbatum* from *Varanus giganteus* from the vicinity of Mt. Conner on the Curtin Springs cattle station, Northern Territory, and a cohort of *A. limbatum* from *Varanus acanthurus* from the Fitzroy River, Kimberly, Western Australia (Fig. 1; Additional file 1: Table S1). Ticks were stored in ethanol at ambient temperature until the current study, and morphologically identified to species, sex, and life stage using standard morphological keys for Australian ticks [18, 19].

### PCR screening of Australian varanid ticks for *Borrelia*

Genomic DNA was extracted from individual ticks using the DNeasy Blood and Tissue Kit (QIAGEN, Germany) following the manufacturer’s protocol. DNA samples were screened for *Borrelia* with nested PCRs targeting the *flaB* and 16S rRNA genes, as previously described [20], using 0.4 M of each primer, 1.5 mM of MgCl_2_ and KAPA Taq DNA polymerase. PCR amplicons were electrophoresed through 1% agarose gels stained with SYBRsafe, and positive amplicons were excised from the gel, purified, and selected samples Sanger sequenced using both 5’ and 3’ PCR primers (Additional file 1: Table S1). Samples were only considered *Borrelia*-positive with consistent amplification of both *flaB* and 16S loci.

### Metagenomic sequencing and assembly of Australian varanid *Borrelia* genomes

Individual representative *Borrelia*-positive tick DNA samples were prepared for shotgun metagenomic sequencing using the Illumina DNA Prep Library Kit and sequenced on a MiSeq using 300 base pair (bp) paired-end chemistry (Illumina, USA) or NextSeq using 150 bp paired-end chemistry at the Genome Discovery Unit—Australian Cancer Research Foundation Biomolecular Resource Facility, The John Curtin School of Medical Research, Australian National University. Metagenomic assembly of *Borrelia* spp. was performed with metaspades [21], with *Borrelia* contigs identified with mmseq2 [22] searching against a custom database of curated *Borrelia* genomes from the National Center for Biotechnology Information (NCBI) Reference Sequence Database (RefSeq). *Borrelia* chromosomal contigs were scaffolded to the *B. turcica* strain IST7 chromosome with Mauve v1.1.1 [23], and contigs were corrected and gaps closed by mapping reads back to scaffolded contigs with minimap2 [24]. Plasmid contigs were checked for completeness and circularisation by aligning plasmids to themselves and searching for terminal overlaps and terminal telomere structures.

### *Borrelia* sp. RT1S and RT5S from *V. salvator*

In addition to Australian varanid *Borrelia* spp., we sequenced *Borrelia* sp. strains RT1S and RT5S that were isolated from *A. varanense* ticks engorged on *V. salvator* in Bogor, West Java, Indonesia, as reported previously [8]. Strains RT1S and RT5S were isolated from separate *A. varanense* salivary glands from the same host animal in BSK-H medium, and genomic DNA was extracted from low passage cultures using the Genomic-tip 100/G kit (QIAGEN, Germany).

*Borrelia* strains RT1S and RT5S were sequenced with Illumina and MinION technologies. DNA was prepared for Illumina sequencing using the Illumina DNA Prep Library Kit and sequenced on a MiSeq using 300 bp paired-end chemistry (Illumina, USA). MinION sequencing libraries were prepared using the Ligation Sequencing Kit [Oxford Nanopore Technologies (ONT), UK], and base calling was performed with Guppy v4.2.2. Raw Illumina and ONT sequences were quality filtered with Fastp v0.23.1 [25] (for Illumina reads), and Porechop v0.2.4 (https://github.com/rrwick/Porechop), Filtlong v0.2.1 (https://github.com/rrwick/Filtlong), and Pacasus v1.2 [26] (for ONT reads). Assembly of ONT reads was performed using Canu v2.2 [27] and Flye v2.8 [28] with five rounds of polishing with Pilon v1.24 [29]. Contigs from different assemblies were aligned together to determine the correctness and completeness of replicon contigs, and putative plasmid contigs were checked for completeness and circularisation acribed above.

### Genome annotation

Plasmids in the final assemblies were named based on their topology [linear plasmid (lp), circular plasmid (cp)] and size (to the nearest kilo base); in cases where multiple plasmids shared similar topology and size, they were suffixed alphabetically (e.g. cp30, cp30-B). Genomic features were annotated using NCBI Prokaryotic Genome Annotation Pipeline v5.2 [30], and InterProScan v5.56–89.0 [31] was used to classify putative replicon partitioning proteins and vmps using the CDD, Pfam, and Superfamily databases [32–34], following the methods of Kneubehl et al. [35] and Kuleshov et al. [36]. In brief, the following gene families correlated with the subsequent Pfam and vmp gene families: cd02038 and cd02042 (PF32), PF01672 (PF49), PF02089 (PF50), PF02414 (PF57/62), PF01441 and SSF63515 [variable small proteins (vsps)] and PF00921 and SSF74748 [variable large proteins (vlps)] [36]. PF32 and PF57/62 plasmid partitioning protein genes were aligned with those from Kneubehl et al. [35] with MAFFT [37] and phylogenetic analyses were performed with IQ-TREE v2.2.0 [38] with 1000 ultrafast bootstrap approximations and near-zero branches collapsed into a polytomy. Alignment and phylogenetic analysis of vlp genes, including non-redundant vlp genes from other *Borrelia* spp., were performed in the same manner. All final genomes were deposited in GenBank BioProject PRJNA774887.

### Comparative, phylogenetic, and pangenome analysis

We compared the gene content and chromosomal and plasmid synteny between varanid *Borrelia* chromosomes by aligning whole chromosomal sequences with Mauve v1.1.1 [23] and MUMmer [39] and aligning orthologous protein coding sequences with MCscan [40]. For pangenome analysis, protein coding sequences were identified in all genomes using Prokka v1.12 [41] and parsed into Roary v3.13 [42], which clustered gene orthologues at ≥ 60% amino acid identity and identified core and accessory genes as well as presence/absence of orthologues among the genomes. Phylogenomic analysis utilized 726 core orthologues identified by Roary v3.13 in 21 *Borrelia* genomes (including those from this study), with individual orthologues aligned with MAFFT, and analysis conducted in IQ-TREE v2.2.0 with proportional edge-linked partition models predicted for each orthologous gene set [43], 1000 bootstrap approximations [44], and gene and site concordance factors calculated for each node [45, 46]. 16S and *flaB* phylogenies were performed in the same manner with IQ-TREE v 2.2.0 with model selection and 1000 bootstrap approximations.

## Results

The following results clearly demonstrate that the *Borrelia* spp. identified in ticks collected from *V. salvator*, *V. varius*, and *V. giganteus* are distinct species. Therefore, for clarity, we herein refer to these *Borrelia* spp. by their proposed names: *Borrelia salvatorii* (from *V. salvator*), ‘*Ca*. Borrelia undatumii*’ *(from *V. varius*), and ‘*Ca*. Borrelia rubricentralis’ (from *V. giganteus*).

### PCR screening of Australian varanid ticks

Collectively, 230 ticks from 38 Australian lizards were screened for *Borrelia* by nested PCR, with 45 ticks returning positive (Additional file 1: Table S1). Overall, 5.95% (5/84) of *Bothriocroton undatum* ticks from *V. varius* in the Burragong Valley were positive for *Borrelia*, including male and female *B. undatum* from two out of 12 individual lizards. *Borrelia* was detected in 43.95% (40/91) of ticks from *V. giganteus*, including male and female *A. calabyi* and *A. fimbriatum*, and nymph *A. limbatum* from 10 of 14 lizards. No ticks were *Borrelia*-positive in the cohort of *A. limbatum* from *V. acanthurus*. Preliminary analysis of selected *flaB* and 16S amplicon sequences indicated that all *Borrelia* from *Bothriocroton undatum* ex *V. varius* and *Amblyomma* spp. ex *V. giganteus* were near-identical (*flaB* nucleotide similarity > 99.6%; 16S nucleotide similarity > 99.8%), with the only nucleotide difference between sequences being single nucleotide polymorphisms, all of which were non-synonymous for *flaB* sequences (data not shown).

### Genome sequencing and assembly

Metagenomic Illumina sequencing and de novo assembly produced complete *Borrelia* chromosomes for both ‘*Ca*. Borrelia undatumii*’* AG58 from *Bothriocroton undatum* ex *V. varius* and ‘*Ca*. Borrelia rubricentralis*’* P9F1 from *A. calabyi* ex *V. giganteus* (0.912 Mb and 0.937 Mb, respectively). Each chromosomal assembly included a maximum of three contigs (minimum length 0.34 Mb), with gaps closed by mapping bridging reads across open spans < 1 kb. Putative plasmid contigs were also identified, with six putative linear plasmid contigs (6–65 kb in length) for ‘*Ca*. B. undatumii*’*, and five linear plasmid contigs (23–74 kb in length) and two complete circular plasmids (32 kb in length) for ‘*Ca*. B. rubricentralis*’* (Table 1). For ‘*Ca*. B. undatumii*’*, plasmid contigs did not represent complete replicons, although telomere structures were identified on the right end of plasmids lp6, lp12, and lp31, confirming their linear structure. Likewise, all linear plasmids from ‘*Ca*. B. rubricentralis’ were incomplete, with no telomere structures identified at either end of the plasmid contigs. ‘*Ca.* B. rubricentralis’ plasmids cp32 and cp32-B were confirmed to be circular by overlapping terminal ends of the contigs, and were the only complete contigs identified in these assemblies. Each putative plasmid sequence in the metagenomic assemblies was represented by one continuous contig. General sequencing statistics for each assembly are presented in Additional file 1: Table S2.

**Table 1:** Summary of assembled *Borrelia* replicons

Replicon	Complete/partial	Accession no.	Length (base pairs)	CDS (no.)	Mean coverage (Illumina/ONT)
*Borrelia salvatorii* strain RT1S					
Chromosome	Complete	CP088943	952,250	864	1359.7/609.6
lp23	Complete	CP088944	23,856	34	3554.4/780.8
lp25	Complete	CP088945	25,673	32	5506.1/595.4
cp27	Complete	CP088946	27,661	28	4285.1/438.8
lp27	Complete	CP088947	25,416	32	5015.2/626.5
lp30	Complete	CP088948	30,113	37	9638.7/1231.5
lp30-B	Partial	CP088949	30,059	34	7106.5/781.2
lp51	Complete	CP088950	51,384	61	2751.7/378.2
lp83	Complete	CP088951	82,803	86	2588.0/369.5
*Borrelia salvatorii* strain RT5S					
Chromosome	Complete	CP088936	942,663	856	1464.0/388.8
lp25	Complete	CP088937	25,456	30	8945.1/838.0
cp27	Complete	CP088938	27,771	29	4716.3/318.8
lp27	Complete	CP088939	27,011	30	7108.5/773.9
lp30	Complete	CP088940	30,932	36	5415.2/573.9
lp44	Partial	CP088941	44,552	52	3422.8/319.1
lp83	Complete	CP088942	83,220	86	2669.2/399.9
‘*Candidatus* Borrelia undatumii’ AG58					
Chromosome	Complete	CP086555	912,448	863	101.3/-
lp6	Partial	CP129596	9,178	4	179.3/-
lp9	Partial	CP129597	12,342	8	151.8/-
lp12	Partial	CP129598	12,681	14	129.9/-
lp17	Partial	CP129599	17,500	22	91.9/-
lp31	Partial	CP129600	37,310	31	179.1/-
lp65	Partial	CP129601	65,850	74	170.2/-
‘*Candidatus* Borrelia rubricentralis’ P9F1					
Chromosome	Complete	CP129407	936,962	851	24.4/-
lp23	Partial	CP129410	23,742	25	21.2/-
lp24	Partial	CP129411	24,219	31	31.3/-
lp25	Partial	CP129412	25,027	26	25.1/-
cp32-B	Complete	CP129409	32,481	39	24.7/-
cp32	Complete	CP129408	32,878	41	36.2/-
lp36	Partial	CP129413	36,508	42	28.7/-
lp74	Partial	CP129414	74,710	80	26.4/-

*lp* Linear plasmid, *cp* circular plasmid,* CDS* coding sequence,* ONT* Oxford Nanopore Technologies

**Fig. 2:**
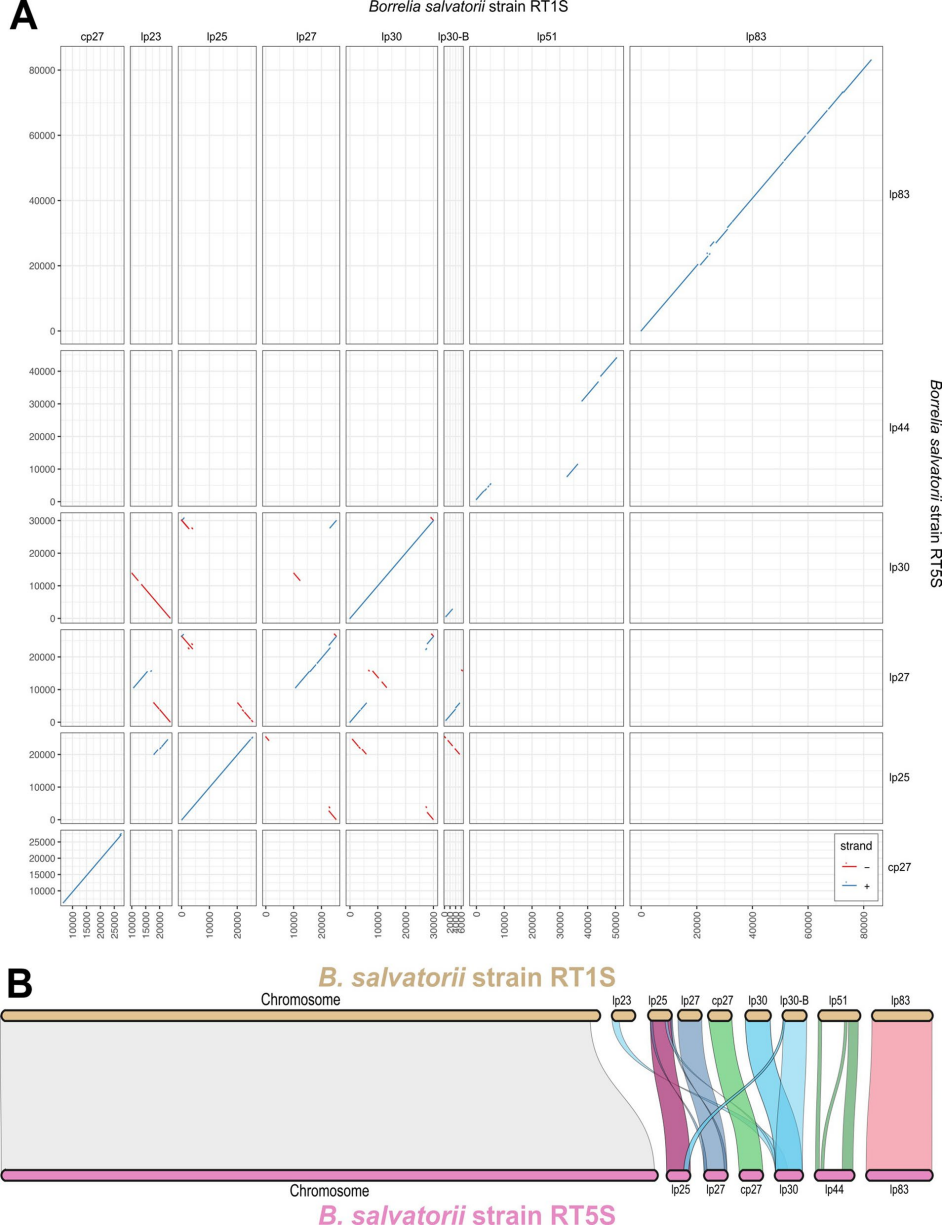
Alignment of *Borrelia salvatorii* strains RT1S and RT5S showing **A** pairwise alignment plots of plasmid sequences with colinear nucleotide regions identified by MUMmer v3.1, and **B** an MCscan karyotype plot showing regions of orthologous macrosynteny between protein coding sequences

In addition to Australian varanid *Borrelia*, we also sequenced two *Borrelia* strains isolated from *A. varanense* ex* V. salvator* in Indonesia [8]. Hybrid de novo assembly of *B. salvatorii* strains RT1S and RT5S from MinION and Illumina sequencing produced a complete chromosome contig for each isolate (9.4–9.5 Mb), and eight and six complete plasmid contigs for strains RT1S and RT5S, respectively (Table 1), including five core plasmids that were highly conserved between strains, and four plasmids that were divergent between strains (Fig. 2).

*Borrelia salvatorii* strains RT1S and RT5S shared five core plasmids: lp83, lp30, lp27, lp25, and cp27. Pairwise alignment of these plasmids to each other showed almost identical gene synteny and nucleotide similarity (83.5–99.5%) between strains (Fig. 2; Additional file 1: Fig. S1). Plasmid cp27, the only circular plasmid identified, had the lowest overall level of average nucleotide identity (83.5%) between strains RT1S and RT5S; however, almost all of this variance occurred in one 5-kb region containing immunogenetic vlps that are known to be highly divergent between strains in other *Borrelia* spp. [47] (Additional file 1: Fig. S1). Both strains RT1S and RT5S contained almost identical (99.5% nucleotide similarity) copies of plasmid lp30; however, strain RT1S contained two divergent copies of lp30 (lp30 and lp30-B) that were almost completely syntenic, but shared only 63.4% nucleotide identity and 56.6% amino acid identity (Fig. 2; Additional file 1: Fig. S1). Both strains contained nearly identical lp27 plasmids, although this plasmid in strain RT1S was actually ~ 25 kb long due to the contraction of two hypothetical proteins and a noncoding region on the right side of the plasmids (Fig. 2; Additional file 1: Fig. S1). *Borrelia salvatorii* strain RT1S lp23 was not present in strain RT5S, although it did share orthologues with genes found on lp30 (Fig. 2; Additional file 1: Fig. S1). In addition, lp51 from strain RT1S and lp44 from strain RT5S shared orthologues at each end of the replicons, but the internal portion of each replicon was highly divergent (Fig. 2; Additional file 1: Fig. S1).

### Phylogenetic analysis

**Fig. 3:**
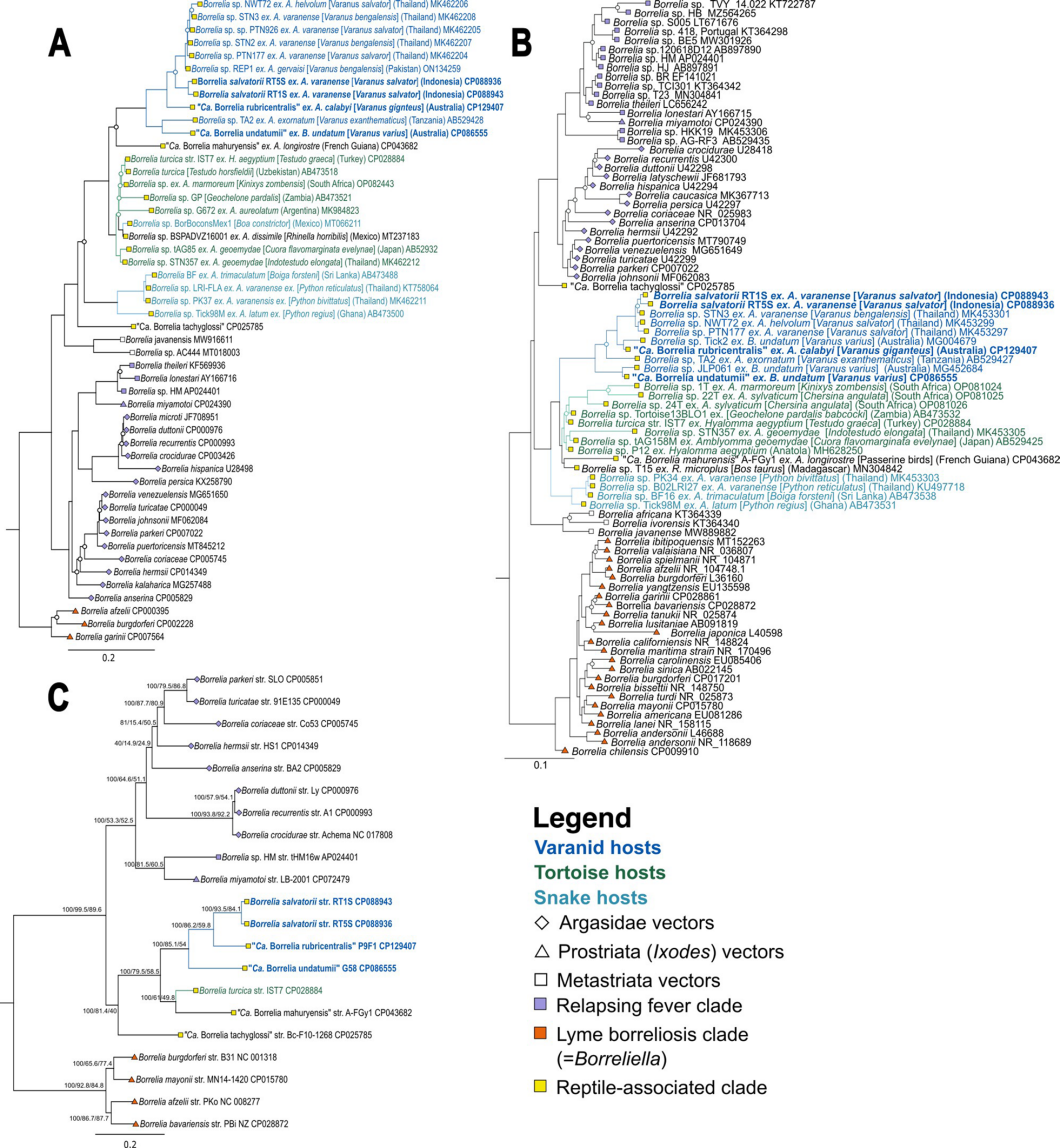
Maximum likelihood phylogenies of reptile-associated (REP) *Borrelia* spp. based on alignment of *flaB* nucleotide sequences (> 301 bp) (**A**), 16S rRNA nucleotide gene sequences (> 1033 bp) (**B**), and 712 shared single copy nucleotide orthologues (**C**). All phylogenies were produced in IQ-TREE v2.2.0 with model selection and 1000 ultrafast bootstrap approximations. Node labels in **A** represent bootstrap support/gene concordance/site concordance; open circle node shapes in **b** and **C** indicate bootstrap support < 70% open circles. *Borrelia* sequences from this study are shown in bold. Root branches are not shown

Maximum likelihood phylogenetic analysis of *flaB* and 16S sequences strongly supports the grouping of *B. salvatorii*, ‘*Ca*. B. undatumii*’*, and ‘*Ca*. B. rubricentralis*’* within a monophyletic varanid-associated clade that includes all previously described varanid genotypes from the Indo-Pacific region. (Fig. 3A–C). In agreement with previous studies, *flaB* and 16S phylogenies placed all REP *Borrelia*, including those from tortoise, snakes, and varanids into a monophyletic clade that is distinct from the hard and soft tick RF clade and the LB clade (Fig. 3A, B). Additionally, within the reptile clade there is strong support for distinct host-associated lineages related to tortoise, snake, and varanid hosts, with the exception of two recently described *Borrelia* spp. from *Boa constrictor* and *Rhinella horribilis* in Mexico [4, 6], which clustered within the tortoise clade (Fig. 3A, B).

Two distinct 16S sequences were previously identified in *B. undatum* from a single individual *V. varius* that differed by 1.5% nucleotide identity [7]. ‘*Ca*. B. rubricentralis’ 16S sequences grouped most closely with one of these sequences, while ‘*Ca*. B. undatumii’ 16S sequences from this study grouped strongly with the other one (Fig. 3B). *Borrelia salvatorii* 16S and *flaB* sequences clustered strongly with sequences from *V. salvator* and *V. bengalensis* from Thailand [5] and Pakistan [12] (Fig. 3A, B).

The results of the phylogenetic analysis based on 712 single copy orthologues also concurred with the 16S and *flaB* phylogenies and with previous whole genome-based *Borrelia* phylogenies [2, 3, 36, 48], and demonstrated overwhelmingly that the REP *Borrelia* clade (with the inclusion of ‘*Ca*. B. mahuryensis’ and ‘*Ca*. B. tachyglossi’) is monophyletic with respect to the RF and LB clades (Fig. 3C). Within the REP clade, all varanid-associated species were monophyletic, with *B. turcica* and ‘*Ca*. B. mahuryensis’ basally positioned to the exclusion of ‘*Ca*. B. tachyglossi’ (Fig. 3C). This suggests that the evolutionary pattern of distinct host-associated REP lineages observed in 16S and *flaB* phylogenies may hold true with the addition of more powerful genomic data. However, genomic data from additional tortoise- and snake-associated *Borrelia* spp. are needed to confirm this hypothesis.

### *Borrelia* chromosomal synteny

Mauve and MCscan alignments demonstrated that varanid *Borrelia* spp. have highly conserved colinear chromosomes, with > 98% of all orthologous protein coding genes in synteny with other *Borrelia* chromosomes. Alignment of whole chromosomes, however, showed several instances of gene insertions and deletions that were synapomorphic for the REP *Borrelia* clade. The insertion of phosphotransferase system lactose/cellobiose transporter subunit IIA, flagellar filament outer layer protein FlaA, and Cof-type HAD-IIB family hydrolase genes (at positions 36,260, 184,184, and 200,384, respectively), and the deletion of a transfer RNA gene [(guanosine-2'-O-)-methyltransferase; at position 41,559] were synapomorphic for the REP clade with the inclusion of ‘*Ca*. B. mahuryensis’ and ‘*Ca*. B. tachyglossi’. In addition, the deletion of chromosome partition protein genes smc and parB, and a putative cytosolic protein gene (at positions 38,399, 455,067, and 133,084, respectively) were synapomorphic for REP *Borrelia* to the exclusion of ‘*Ca*. B. tachyglossi’. Finally, the only synapomorphies specific to varanid *Borrelia* spp. were the deletion of the DNA-3-methyladenine glycosylase gene (position 444,418) from the rRNA operon and the insertion of a small hypothetical protein (position 773,872) (Additional file 1: Fig. S2). The rRNA operon of all varanid *Borrelia* spp. contained three single copy rRNA genes (5S, 23S, 16S) and seven protein-coding genes, including the horizontally acquired putative maltose metabolism genes *glvA* and *glvC*, which are also found in *B. turcica* and ‘*Ca*. B. tachyglossi’ and other REP species, but are absent from all other *Borrelia* spp. [3, 10]. All varanid genomes also included a homologue of the RF antigenic protein glpQ, which is a major immunogenic protein expressed during RF *Borrelia* infection. All of the nucleotide positions given above refer to nucleotide positions along the *Borrelia* sp. RT1S chromosome (CP088943).

### Plasmid sequence analysis

**Fig. 4:**
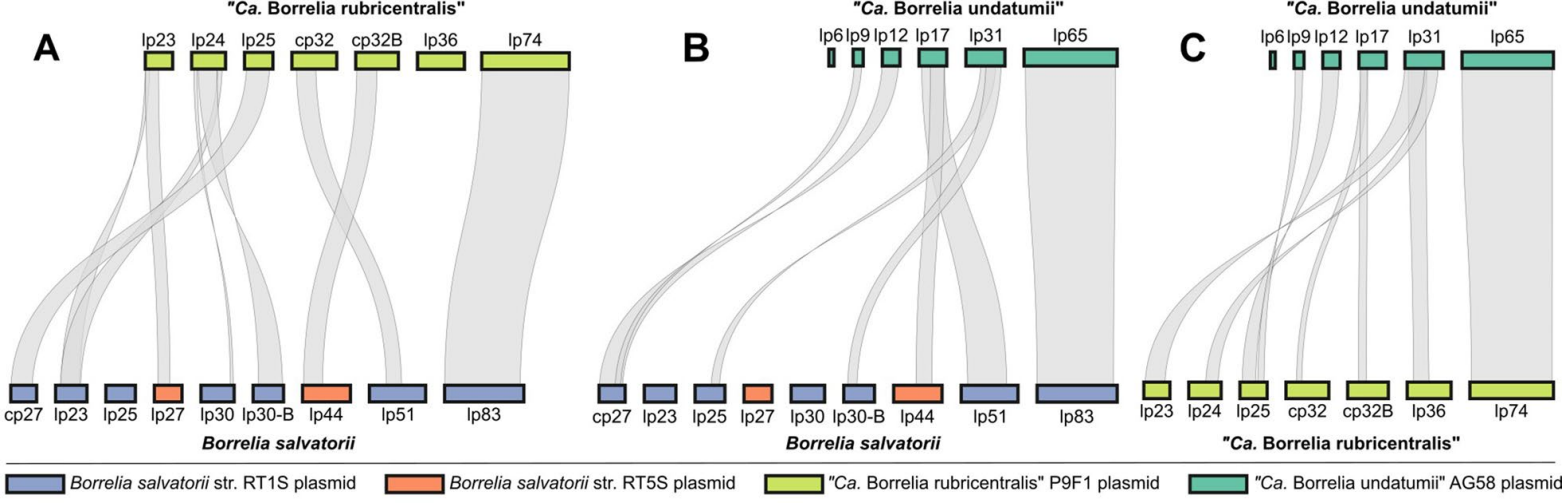
MCscan karyotype plots showing regions of orthologous macrosynteny of protein coding sequences between *Borrelia salvatorii* and ‘*Ca*. Borrelia rubricentralis’ (**A**), *B. salvatorii* and ‘*Ca*. Borrelia undatumii’ (**B**), and ‘*Ca*. B. rubricentralis’ and ‘*Ca.* *Borrelia* *undatumii*' (**C**) plasmids represent the complete set of plasmids present in both strains RT1S and RT5S

Plasmids from *B. salvatorii*, ‘*Ca*. B. undatumii’, and ‘*Ca*. B. rubricentralis’ had an overall low degree of structural conservation between species, but a high degree of shared orthologous gene content, with 57% of plasmid-encoded proteins shared between all three species. Some plasmids were conserved between species, such as lp30-B and lp24 from *B. salvatorii* and ‘*Ca*. B. rubricentralis’, respectively, and cp27, lp25, and lp12 from *B. salvatorii*, ‘*Ca*. B. rubricentralis’, and ‘*Ca*. B. undatumii’, respectively (Fig. 4). The large linear plasmids of each species (lp83, lp74, and lp65) were also highly conserved, not only between *B. salvatorii*, ‘*Ca*. B. rubricentralis’, and *‘Ca*. B. undatumii’, but also with the large linear plasmids of RF species (Additional file 1: Fig. S3).

All REP *Borrelia* also contained linear or circular plasmids (or plasmid contigs) that are related to the LB cp26 plasmid, which is essential for LB *Borrelia* survival (Additional file 1: Fig. S4). The complete REP cp26-like plasmids analysed (*B. salvatorii* cp27, *B. turcica* lp35) contained three highly syntenic but structurally inverted homologous regions to LB cp26 plasmids, which included homologous genes including resT, which is essential for successful replication of linear replicons (Additional file 1: Fig. S4). Varanid *Borrelia*, however, lacked an identifiable variable tick protein/ospC homolog on cp26, which has been shown to be important for host and vertebrate infection, while *B. turcica* lp35 does contain a variable tick protein/ospC homolog. The gene content and ubiquitous nature of cp26-like plasmids in REP *Borrelia* suggest that, like in their LB homologues, they are essential for *Borrelia* survival.

Plasmid partitioning genes are essential for the inheritance and maintenance of plasmids, and five paralogous gene families (PF) have been identified as putative plasmid partitioning genes in *Borrelia*: PF32, PF49, PF50, and P57/62 [13, 49]. These genes are also used to classify plasmid compatibility for LB, and have been used for some RF groups [13, 35, 36]. We identified putative plasmid partitioning genes in our assemblies, as well as the other REP species *B. turcica*, ‘*Ca*. B. tachyglossi’, and ‘Ca. B. mahuryensis’, and compared the PF32 and PF57/62 loci to determine the relationships of REP *Borrelia* plasmids. Because the plasmid assemblies from ‘*Ca*. B. undatumii’ and ‘*Ca*. B. rubricentralis’ are incomplete, we did not identify any putative plasmid partitioning genes in three plasmids from ‘*Ca*. B. undatumii’ (lp17, lp12, and lp6). All other plasmids contained at least one PF57/62 locus, with *B. salvatorii* strain RT5S lp51 containing two distinct PF57/62 copies (Additional file 1: Fig. S5).

**Fig. 5:**
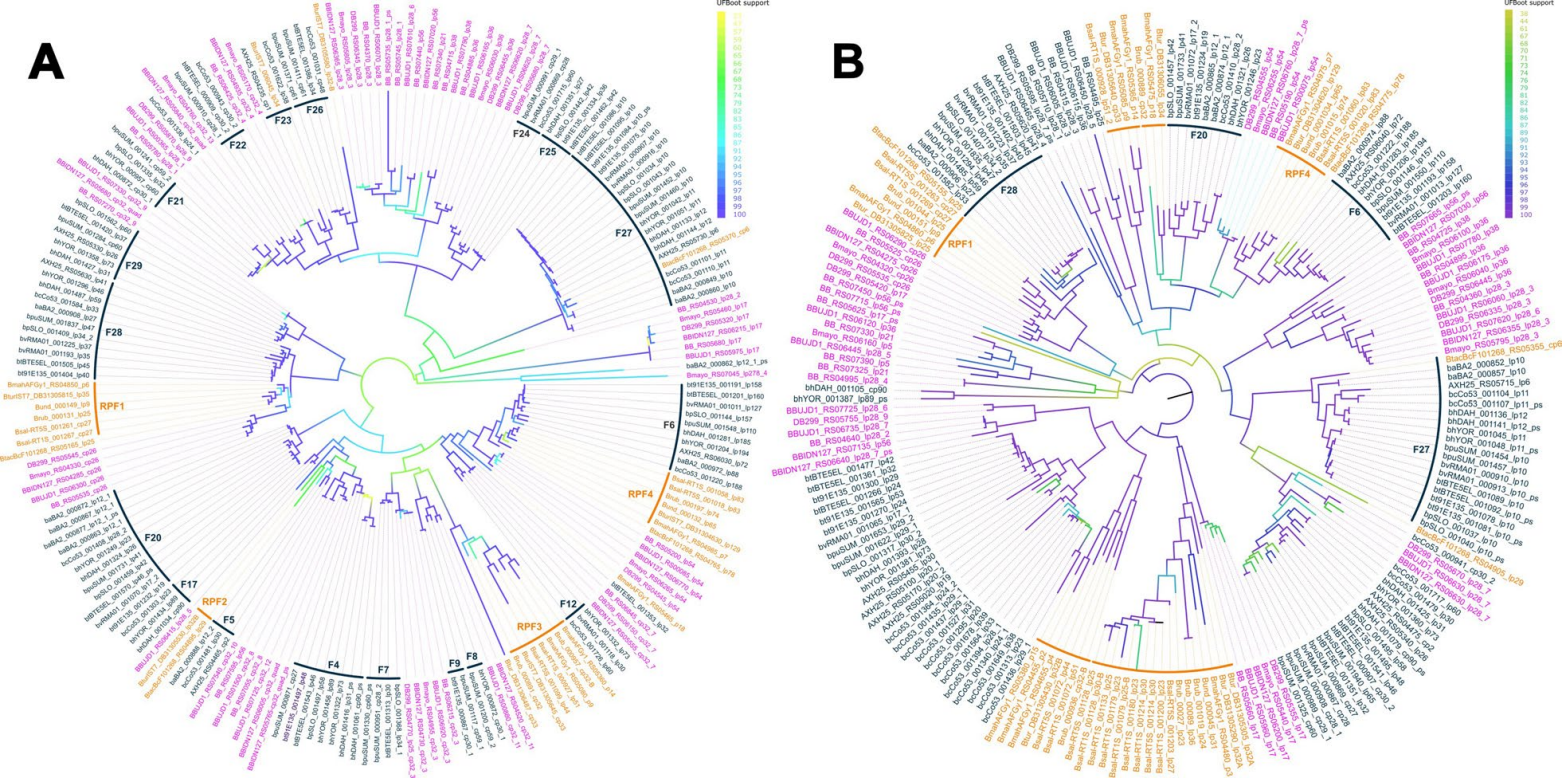
Maximum likelihood phylogenies of REP *Borrelia* plasmids based on PF32 (**A**) and PF57/62 (**B**) nucleotide sequences among a subset of Lyme borreliosis (LB) and relapsing fever (RF) sequences as used by Jones et al. [31] to define RF plasmid compatibility groups. Branches are colour coded according to bootstrap support and taxa labels are colour coded according to their phylogenetic lineage [cyan (LB), purple (RF), orange (REP)]. The outer rings in **A** show the plasmid families based on analysis of PF32 sequences, with purple rings indicating RF plasmid families (F4-29) as defined by Jones et al. [31] and orange rings indicating REP plasmid families (RPF1-4) as defined by our analysis. Labelled outer rings in **B** show plasmid families that are concordant between PF32 and PF57/72 loci, and unlabelled outer rings indicate putative REP plasmid families that were disconcordant with PF32 loci or for which no PF32 loci existed. The isolate prefixes, gene locus, and plasmid are given for each PF32 and PF57/62 locus. See Supplementary Information for details of isolate prefixes

Phylogenetic and clustering analysis of PF32 loci identified four main REP plasmid clusters, and four orphan REP replicons that did not cluster with other REP plasmids. Two orphan PF32 loci from *B. turcica* lp34 and ‘*Ca*. B. tachyglossi’ clustered within known RF plasmid families (F23 and F27, respectively, as defined by Kneubehl et al. [35]). *Borrelia turcica* lp32-B was closely related to the RF F26 plasmid family, while ‘*Ca*. Borrelia mahuryensis’ lp54 PF32 was within a polyphyletic group containing LB lp54 and cp32 plasmids as well as *Borrelia turicatae* BTE5EL lp32 (Fig. 5A). The remaining REP PF32 loci formed four distinct monophyletic clusters, designated REP plasmid families (RPF) 1-4, with RPF1 associated with LB cp26 plasmids, RPF2 positioned next to RF F5 plasmids, RPF3 positioned next to RF F8-9 and LB cp32 plasmids, and RPF4 representing the long linear megaplasmids and positioned sister to the RF F6 megapasmids and LB lp54 plasmids (Fig. 5A).

As many plasmids lacked a PF32 loci, we also used the PF57/62 loci, which were identified on all plasmids except those that lacked any putative plasmid partitioning genes (likely due to incompleteness). Phylogenetic analysis of REP PF57/62 genes had limited congruency with the PF32 analysis; however, it demonstrated an agreement with the PF32 analysis that most REP plasmids form monophyletic lineages that are distinct from RF and LB plasmid families. There was broad congruency between the RPF1 and RPF4 families between the two phylogenies, indicating a more stable inheritance pattern for these plasmids throughout all *Borrelia*, including REP species (Fig. 5B). Other REP PF57/62 loci clustered into four distinct monophylies, although these clusters did not concur with the PF32 phylogeny (Fig. 5). These groups included two clades that were sister to RF F20 plasmids, one clade that was sister to LB lp17 plasmids, and one clade that was sister to a large cluster of unclassified RF plasmids (Fig. 5B). In addition, two orphan PF57/62 genes were identified from ‘*Ca*. B. tachyglossi’, with one from cp6 clustering with RF F27, in agreement with the PF32 analysis.

We also investigated the phylogenetic relatedness of REP vlp to the immunogenic vlp and vsp proteins of RF species which underpin their complex polyphasic antigenic switching phenotype. Immunogenic vlps loci were found on plasmids cp27, lp25, and lp12 from *B. salvatorii, ‘Ca*. B. rubricentralis’, and ‘*Ca*. B. undatumii’, respectively, although they had low (< 44%) levels of homology to RF vlp amino acid sequences, and even less to LB vlp homologs. No vsp sequences were identified. Unlike their homologues in RF species, REP vlp sequences were highly reduced in number, with a maximum of five vlps found on individual plasmids. *B. salvatorii* cp27 contained five vlp loci, ‘*Ca*. B. rubricentralis*’* lp25 contained five vlp loci, and ‘*Ca*. B. undatumii’ contained just two vlp loci (Additional file 1: Fig. S5). *‘Ca*. B. rubricentralis*’* lp25, *B. salvatorii* cp26, and *B. turcica* lp35 all contained clusters of 5–11 vlps which cantered on a homologous vlp1 sequence (Additional file 1: Fig. S6). This vlp1 may represent a putative expression site for antigenic switching; however, there was limited homology between REP vlp1 and known vlp expression sites in RF and LB species, and upstream and downstream homology sequences were not identified. No ospC, vtp, or vlsE homologs were identified in varanid *Borrelia*. Phylogenetic analysis indicated that all varanid vlp proteins clustered together into a single monophyletic cluster in which vlps from individual species largely grouped together into distinct clades (Additional file 1: Fig. S7). This is in stark contrast to the vlp sequences of RF *Borrelia* which were highly variable and paraphyletic across the phylogeny (Additional file 1: Fig. S7).

### Pangenome

**Fig. 6:**
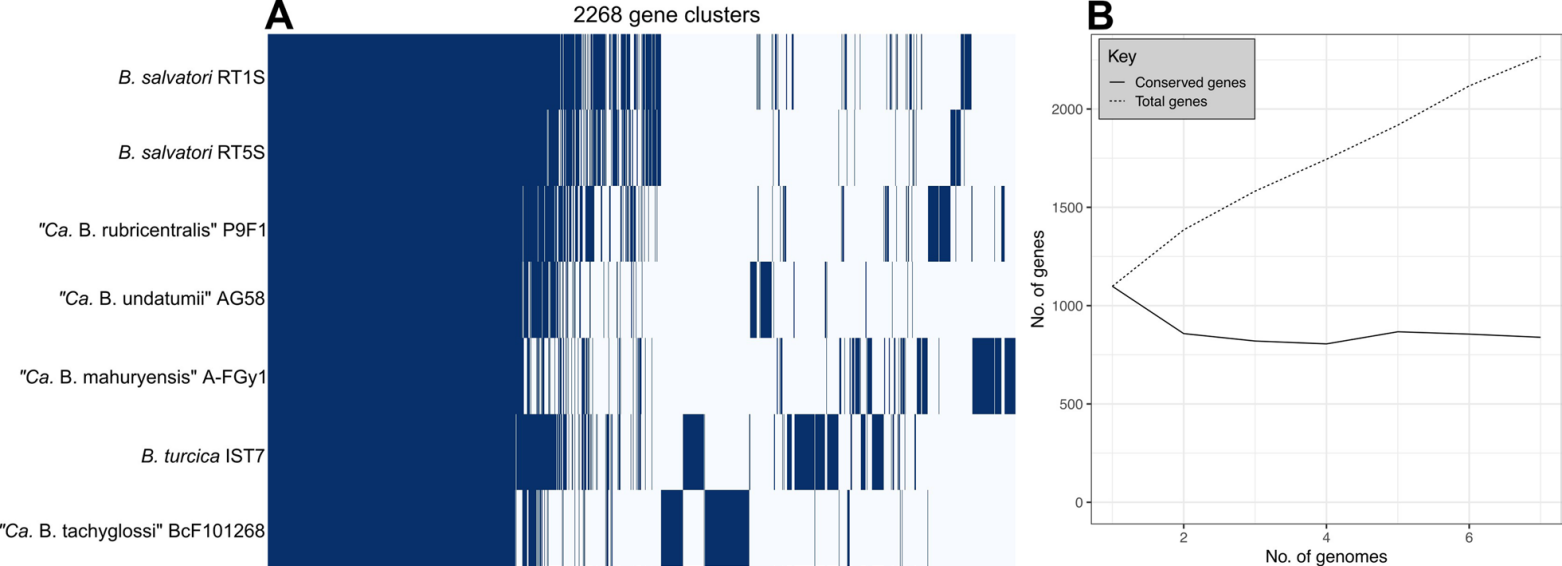
Pangenome matrix showing the presence/absence of 2268 orthologues among REP *Borrelia* species created with Roary v3.11.2 (**A**) and graph of the number of core genes and total genes as pangenome size increases (**B**)

Pangenome analysis included all available REP *Borrelia* genomes, including *B. turcica*, ‘*Ca*. B. mahuryensis’, and ‘*Ca*. B. tachyglossi’, in addition to the four genomes sequenced in this study (Fig. 6A). Pangenome analysis identified a core genome of 839 gene clusters that were present in at least six of the seven genomes, and an accessory genome of 1429 gene clusters that were present in less than six of the seven genomes. While the number of core genes stabilized quickly after the addition of just three genomes to the pangenome, the accessory genome continued to increase with each additional genome (Fig. 6B). This has been observed previously for other *Borrelia* groups and is likely attributable to the high degree of plasmid diversity and numerous paralogous gene families characteristic of *Borrelia* genomes [35]. The pangenome matrix illustrates the pattern of gene presence/absence among REP *Borrelia* and demonstrates that there is minimal sharing of accessory gene clusters between species, and that distinct blocks of accessory genes are differentially present between species, and even between *B. salvatorii* strains (Fig. 6A). These differentially present gene blocks are likely linked to diverse plasmids that contain unique gene content between species, and may represent genetic elements important for host-specific adaptations.

### Proposed candidate names

Due to their unique genomic properties, vertebrate and tick associations, and phylogenetic distinctness as described above, we propose the designations *Borrelia salvatorii*, ‘*Candidatus* Borrelia undatumii’, and ‘*Candidatus* Borrelia rubricentralis’, for these novel putative species identified in ticks from *V. salvator*, *V. varius*, and *V. giganteus*, respectively. The specific designations relate to (1) the putative host species of *B. salvatorii*, i.e.* V. salvator*; (2) the putative tick vector of ‘*Ca.* Borrelia undatumii’, i.e. *Bothriocroton undatum*; and (3) the ‘red centre’, which describes the arid inland desert of Australia in which *V. giganteus* (the putative host of ‘*Ca.* Borrelia rubricentralis’) occurs.

## Discussion

We describe herein the genomes of three novel *Borrelia* species associated with *Varanus* hosts and provide the first genomic resources for this unique *Borrelia* clade. Although the first varanid *Borrelia* was identified over a decade ago from *Amblyomma exornatum* ex *V. exanthematicus* [9], only recently have molecular studies begun uncovering their true diversity, with at least 10 distinct genotypes now known from throughout the Indo-Pacific region. However, characterisation of these genotypes has relied largely on 16S rRNA and *flaB* gene sequence analysis, and whether indeed these genotypes all constitute putative species is unresolved, and thus requires the analysis of additional genomic data.

We also sequenced two strains of *B. salvatorii*, *B. salvatorii* RT1S and *B. salvatorii* RT5S, that were isolated from the salivary glands of separate *A. varanense* ticks engorged on the same *V. salvator* host [8]. The plasmid content of these two strains differed markedly, although both strains contained a core genome consisting of five plasmids (lp83, lp30, lp27, lp25, and cp27). Furthermore, duplicate copies of lp30 were found in strain RT1S, and although these plasmids are highly syntenic they are unusually dissimilar at both the nucleotide and amino acid level, although no pseudogenes were identified in either. This level of variation in plasmid content between strains is not unusual for *Borrelia*, and high levels of variation and structural plasticity between closely related strains are common for most RF and LB species. In comparison, the chromosomes of *B. salvatorii*, and all other REP *Borrelia*, were remarkably stable and syntenic with those of other *Borrelia* spp.

Pangenome analysis demonstrated that the REP pangenome had 2268 gene clusters, and was thus smaller than the RF pangenome (2576–2514 gene clusters), but larger than the LB pangenome (~ 1859 gene clusters) [35, 50, 51]. However, analysis of RF genomes likely overestimates the number of gene clusters due to the large number of paralogous gene clusters and high numbers of paralogous vsp and vlp genes [35]. Overall, REP *Borrelia* had a relatively closed pangenome with most genes shared between most isolates, and relatively few species-specific genes, with the majority of accessory genes inherited in blocks, which likely represent coinherited sets of genes on plasmids.

Classification of LB plasmid compatibility groups has largely relied on the analysis of PF32 loci [13]. Initial attempts to apply this methodology to RF species were successful and demonstrated that there exists a mix of shared and unique plasmid types between LB and RF clades [35, 36]. The results of our phylogenetic analysis of REP plasmids based on PF32 and PF57/62 genes are similar to those of Kneubehl et al. [35], and demonstrate that REP *Borrelia* contains at least four distinct plasmid families based on PF32 loci (designated RPF1-4), and perhaps as many as six based on PF57/62 loci. Our analysis indicated that while most REP plasmids are distinct from those of LB and RF, there exists a shared ancestry between them, which likely represents core genomic elements that are required for survival. For example, sections of the PF32 phylogeny representing the cp26/RPF1/F28 and lp54/RPF4/F6 subtrees mirror the whole genome-based phylogeny presented in Fig. 3 indicating the shared inheritance of these genomic elements with the chromosome. In addition, a limited number of orphan REP plasmids clustered within RF plasmid families, and may be a result of more recent horizontal gene transfer.

Many REP plasmids were found to lack PF32 genes (some through incomplete assembly), and analysis showed that PF57/62 loci had limited phylogenetic congruency with PF32loci but demonstrated a similar general patterns of plasmid relatedness to the PF32 phylogeny, with most REP plasmids clustering into distinct REP-specific clades. This was also noted by Kneubehl et al. [35] in their analysis of RF plasmids, and although analysis of PF57/62 loci somewhat assists the classification of these plasmids, a more a robust systematic methodology is likely needed to resolve the relationship between REP and RF *Borrelia* plasmids. The incongruence between PF32 and PF57/62 phylogenies is likely rooted in the rapid primordial diversification and functional conservation of PF32 genes, which provides suitable genetic variability for phylogenetic analysis, compared to PF57/62 genes, which have much higher levels of genetic variability that may obscure a phylogenetic signal [52].

The vlp and vsp genes identified in REP *Borrelia* had limited homology to those of RF species, and were also severely reduced in overall number and in their amino acid diversity. In each genome, vlps were clustered on one plasmid, with the cluster beginning with vlp1 that was homologous in *B. turcica*, *B. salvatorii*, and ‘*Ca*. B. rubricentralis’. Conservation of vlp1 between REP species suggest that it may be an expression site for the expression of other vlps. However, there was limited homology between vlp1 and known RF and LB vlp expression sites, and upstream and downstream homology sequences were not identified. Further analysis of REP vlp is needed to resolve the antigen switching mechanisms of REP *Borrelia*. Phylogenetic analysis of vlp amino acid sequences (Additional file 1: Fig. S7) also demonstrated that REP *Borrelia* vlps cluster into one monophyletic group and that vlps from each REP species cluster in a species-specific manner.

The role of varanids as true biological reservoirs of *Borrelia* has not been established, as all *Borrelia* isolates characterised to date have been from engorged ticks, including *Amblyomma varanense*, *Amblyomma helvolum*, *Amblyomma gervaisi*, *Amblyomma exornatum*, *Amblyomma calabyi*, *Amblyomma fimbriatum*, *Amblyomma limbatum*, and *Bothriocroton undatum*. Likewise, the role of these ticks as true *Borrelia* vectors is unknown, and while all of these tick species are reptile specialists (and some of them *Varanus* specialists), they often parasitize multiple reptile species, including snakes, varanids, and other squamates [53, 54]. Nevertheless, there appears to be a high level of host adaptation among these *Borrelia*. For example, although several varanid- and snake-associated *Borrelia* genotypes have been identified in *A. varanense* ticks from different hosts, including *V. salvator*, *V. bengalensis*, *Python bivittatus*, and *Python reticulatus* [5, 11], varanid-associated *Borrelia* genotypes have never been recovered from *A. varanense* collected from snakes, or vice versa.

Moreover, the phylogenomic and phylogenetic analyses presented here and previously [5, 8] all demonstrate convincingly that varanid-associated *Borrelia*, in addition to tortoise-associated and snake-associated *Borrelia*, separately group into distinct monophyletic clades within the REP lineage. This pattern is indicative of a high level of host adaptation and *Borrelia*-host coevolution within the REP lineage, which is not observed in other *Borrelia* lineages, where other evolutionary forces are hypothesized to drive speciation [55–57].

The data presented here suggest that varanid-associated *Borrelia* likely occur throughout the entire geographic range of the Varanidae, which encompasses much of central and southern mainland Asia; the Middle East; Africa; the Malay Archipelago, including Indonesia and Papua New Guinea; and Australia [58]. Although *Varanus* is hyperdiverse in Australia compared to other biogeographic regions [58], molecular evidence indicates an Asian origin for the Varanidae, with dispersal to Australia ca. 39–26 million years ago via dispersal events before the collision of Australian and Asian landmasses [59]. Given this biogeographic scenario, it seems probable that ancestral *Varanus-*associated *Borrelia*, and indeed perhaps also ancestral *Amblyomma* spp., colonized Australia at the same time. With > 74 species of *Varanus* worldwide, and 30 in Australia alone, there are undoubtedly numerous additional *Varanus*-associated *Borrelia* species and genotypes awaiting discovery.

## Conclusions

REP *Borrelia* were once considered biological peculiarities and gained little scientific attention compared to their more enigmatic disease-causing cousins. However, novel REP *Borrelia* are increasingly being discovered around the world in association with new hosts and putative metastriate tick vectors. We now understand that REP *Borrelia* represents a distinct lineage that is evolutionarily, ecologically, and genetically unique compared to LB and RF *Borrelia*. This work addresses important gaps in genomic knowledge of REP *Borrelia* and provides crucial resources that will likely underpin the investigation of important evolutionary hypotheses regarding *Borrelia* evolution. This work has doubled the number of publicly available REP *Borrelia* genomes, including the first from *Varanus* hosts, and two strains of the same species, *B. salvatorii*. Through our investigation of intra- and interspecies genomic diversity among all available REP genomes, including plasmid diversity and relatedness, we have produced, to the best of our knowledge, the most complete phylogenetic analysis to date of REP *Borrelia*. Collectively, these genomic resources and our comparative characterisation of these novel REP genomes are likely foundational for future investigations designed to untangle the complex evolutionary history of *Borrelia*, and have important implications for our understanding of *Borrelia*-tick coevolution, host adaptation, and the ecological drivers of speciation.

### Supplementary Information

**Additional file 1: Table S1**. Summary of Australian varanid tick samples tested for *Borrelia*. **Fig. S1**. Pairwise alignment plots of *Borrelia salvatorii* strains RT1S and RT5S core plasmids (**A**), accessory plasmids (**B**), and duplicated plasmids (**C**). **Fig. S2**. Schematic alignment of the ribosomal RNA operon of relapsing fever (RF), reptile-associated (REP), and Lyme borreliosis (LB) *Borrelia* highlighting the deletion of the *mag* gene, which is synapomorphic for varanid-associated *Borrelia* spp. **Fig. S3**. MCscan karyotype orthologous protein coding genes between the conserved large linear megaplasmids of *Borrelia salvatorii* and ‘*Candidatus* (*Ca*.) Borrelia rubricentralis’, ‘*Ca*. Borrelia undatumii’, *Borrelia turcica*, ‘*Ca.* Borrelia tachyglossi’, *Borrelia miyamotoi*, and *Borrelia hermsii*. These large linear megaplasmids appear to be highly conversed among all REP and RF *Borrelia*. **Fig. S4**. MCscan plot showing orthologous protein-coding genes between *Borrelia burgdorferi* B31 plasmid cp26 and *Borrelia turcica* IST7 plasmid lp25 and *Borrelia salvatorii* RT1S cp27. **Fig. S5**. Schematic of varanid-associated *Borrelia* spp. and the approximate locations of putative plasmid partitioning genes, and variable large proteins. **Fig. S6**. Schematic gene map of vlp gene clusters in REP *Borrelia* replicons, including vlp1 (green), which was homologous in all genomes. **Fig. S7**. Phylogenetic reconstruction of REP and RF *Borrelia* vlp amino acid sequences. Extended caption for Fig. 5. The isolate, open reading frame number, and plasmid name is given for each sequence.

## Data Availability

All genomic data are available through NCBI BioProjects PRJNA783659 and PRJNA774887.
