# Supplementary material for: Characterisation and comparative genomics of three new Varanus-associated Borrelia spp. from Indonesia and Australia
Source: Parasit Vectors. 2023 Sep 5;16:317. doi: 10.1186/s13071-023-05937-4 (PMC10481545; doi:10.1186/s13071-023-05937-4)
Supplement: Supplementary file 1 — Additional file 1: Table S1. Summary of Australian varanid tick samples tested for Borrelia. Fig. S1. Pairwise alignment plots of Borrelia salvatorii strains RT1S and RT5S core plasmids (A), accessory plasmids (B), and duplicated plasmids (C). Fig. S2. Schematic alignment of the ribosomal RNA operon of relapsing fever (RF), reptile-associated (REP), and Lyme borreliosis (LB) Borrelia highlighting the deletion of the mag gene, which is synapomorphic for varanid-associated Borrelia spp. Fig. S3. MCscan karyotype orthologous protein coding genes between the conserved large linear megaplasmids of Borrelia salvatorii and ‘Candidatus (Ca.) Borrelia rubricentralis’, ‘Ca. Borrelia undatumii’, Borrelia turcica, ‘Ca. Borrelia tachyglossi’, Borrelia miyamotoi, and Borrelia hermsii. These large linear megaplasmids appear to be highly conversed among all REP and RF Borrelia. Fig. S4. MCscan plot showing orthologous protein-coding genes between Borrelia burgdorferi B31 plasmid cp26 and Borrelia turcica IST7 plasmid lp25 and Borrelia salvatorii RT1S cp27. Fig. S5. Schematic of varanid-associated Borrelia spp. and the approximate locations of putative plasmid partitioning genes, and variable large proteins. Fig. S6. Schematic gene map of vlp gene clusters in REP Borrelia replicons, including vlp1 (green), which was homologous in all genomes. Fig. S7. Phylogenetic reconstruction of REP and RF Borrelia vlp amino acid sequences. Extended caption for Fig. 5. The isolate, open reading frame number, and plasmid name is given for each sequence. [file 13071_2023_5937_MOESM1_ESM.pdf]

Additional file 1

Table S1. Summary of Australian goanna tick samples tested for *Borrelia*

Goanna species	Goanna ID	Tick species	Tick life stage	Number	Positive for *Borrelia* (%)
Varanus varius (NSW)	1-11	*B. undatum*	Female	4	1 (25%)*
		B. undatum	Male	11	2 (18.18%)*
	2-11	*B. undatum*	Female	4	1 (25%)*
		B. undatum	Male	14	1 (7.14%)*
	4-11	*B. undatum*	Female	5	0
		B. undatum	Male	3	0
	5-11	*B. undatum*	Female	5	0
		B. undatum	Male	17	0
	7-11	*B. undatum*	Female	1	0
		B. undatum	Male	5	0
	9-11	*B. undatum*	Male	1	0
		B. undatum	Nymph	7	0
	10-11	*B. undatum*	Female	4	0
		B. undatum	Male	1	0
	1-17	*B. undatum*	Female	1	0
	3-17	*B. undatum*	Female	1	0
	4-17	*B. undatum*	Nymph	1	0
	7-17	*B. undatum*	Male	1	0
	8-17	*B. undatum*	Male	1	0
			**Total**	**84**	**5 (5.95%)**
Varanus giganteus (NT)	P9	*A. calabyi*	Female	15	4 (26.67%)*
		A. calabyi	Male	10	2 (20%)
		A. limbatum	Nymph	2	0
	P11	*A. limbatum*	Nymph	2	0
	P12	*A. calabyi*	Male	2	1 (50%)*
	P14	*A. calabyi*	Female	1	1 (100%)*
	P20	*A. calabyi*	Female	1	0
	P22	*A. calabyi*	Female	2	0
		A. fimbriatum	Female	4	2 (50%)*
	P26	*A. calabyi*	Female	3	0
		A. fimbriatum	Female	1	1 (100%)*
		A. limbatum	Nymph	12	4 (33.33%)*
	P27	*A. calabyi*	Female	1	1 (100%)
	P28	*A. calabyi*	Female	1	1 (100%)
		A. fimbriatum	Female	7	5 (71.43%)
		A. fimbriatum	Male	12	11 (91.67%)*
	P31	*A. calabyi*	Female	1	1 (100%)
		A. calabyi	Male	5	2 (40%)
	P34	*A. calabyi*	Female	2	2 (100%)*
		A. fimbriatum	Female	2	1 (50%)
	P36	*A. calabyi*	Female	2	1 (50%)*
	P37	*A. calabyi*	Male	1	0
	P38	*A. limbatum*	Nymph	2	0
			**Total**	**91**	**40 (43.95%)**
V. acanthurus (WA)	VA01	*A. limbatum*	Male	7	0
		A. limbatum	Female	1	0
	VA02	*A. limbatum*	Male	7	0
	VA03	*A. limbatum*	Male	5	0
	VA04	*A. limbatum*	Male	5	0
	VA05	*A. limbatum*	Male	2	0
	VA06	*A. limbatum*	Male	2	0
	VA07	*A. limbatum*	Male	7	0
	VA08	*A. limbatum*	Male	3	0
	VA09	*A. limbatum*	Male	3	0
	VA11	*A. limbatum*	Male	2	0
	VA12	*A. limbatum*	Male	3	0
		A. limbatum	Female	1	0
	VA15	*A. limbatum*	Male	7	0
			**Total**	**55**	**0**

* Indicates samples for which *flaB* and 16S rRNA amplicons were sequenced.

Table S2. Sequencing statistics for metagenomic sequencing of *Borrelia*

Tick ID	Tick species	Host ID	Host species	Sequencing platform	Read length	Reads passing QC	*Borrelia* reads	% *Borrelia* reads
AG58	*Bot. undatum*	2-11	*V. varius*	Illumina NextSeq	2 x 150 bp	614,334,544	822,237	0.13%
P9F1	*A. calabyi*	P9	*V. giganteus*	Illumina MiSeq	2 x 300 bp	37,758726	925,977	2.45%

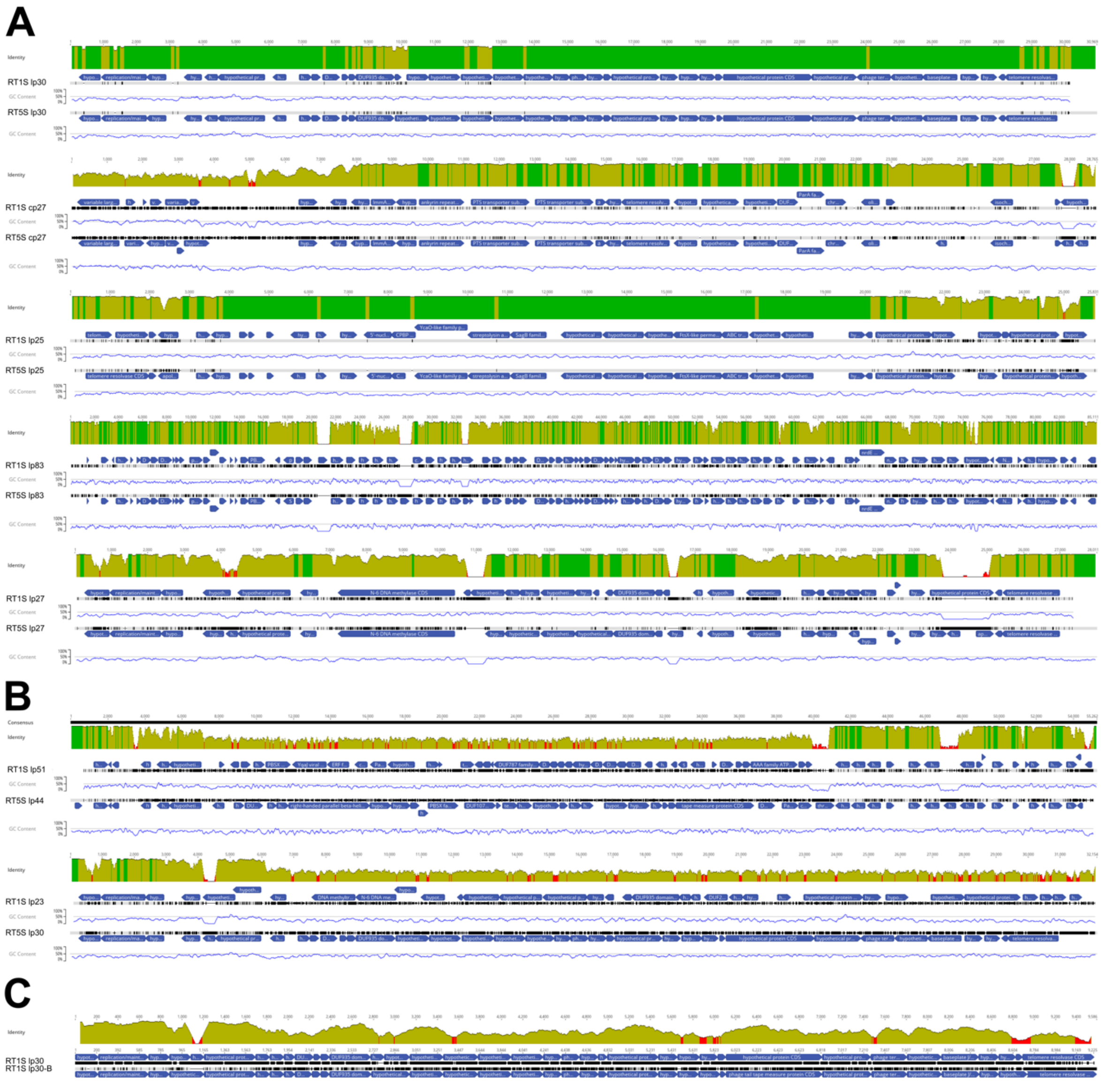

Figure S1 Pairwise alignment plots of *Borrelia salvatorii* strains RT1S and RT5S core plasmids (A), accessory plasmids (B), and duplicated plasmids (C). Pairwise identity plots (log scale, 99 nt sliding window) are shown above each alignment, protein coding sequences are annotated in purple, and GC% plots shown below each alignment.

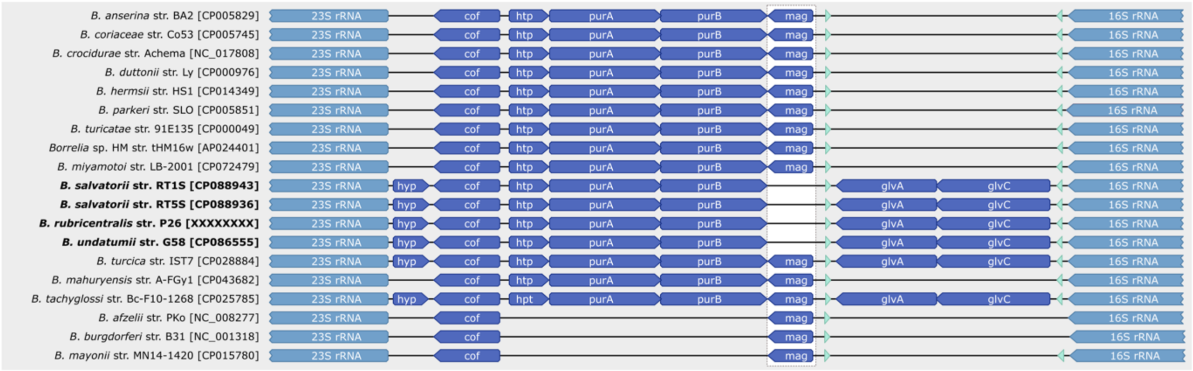

Figure S2 Schematic alignment of the ribosomal RNA operon of RF, REP, and LB *Borrelia* highlighting the deletion of the *mag* gene, which is synaptomorphic for Varanid-associated *Borrelia* spp.

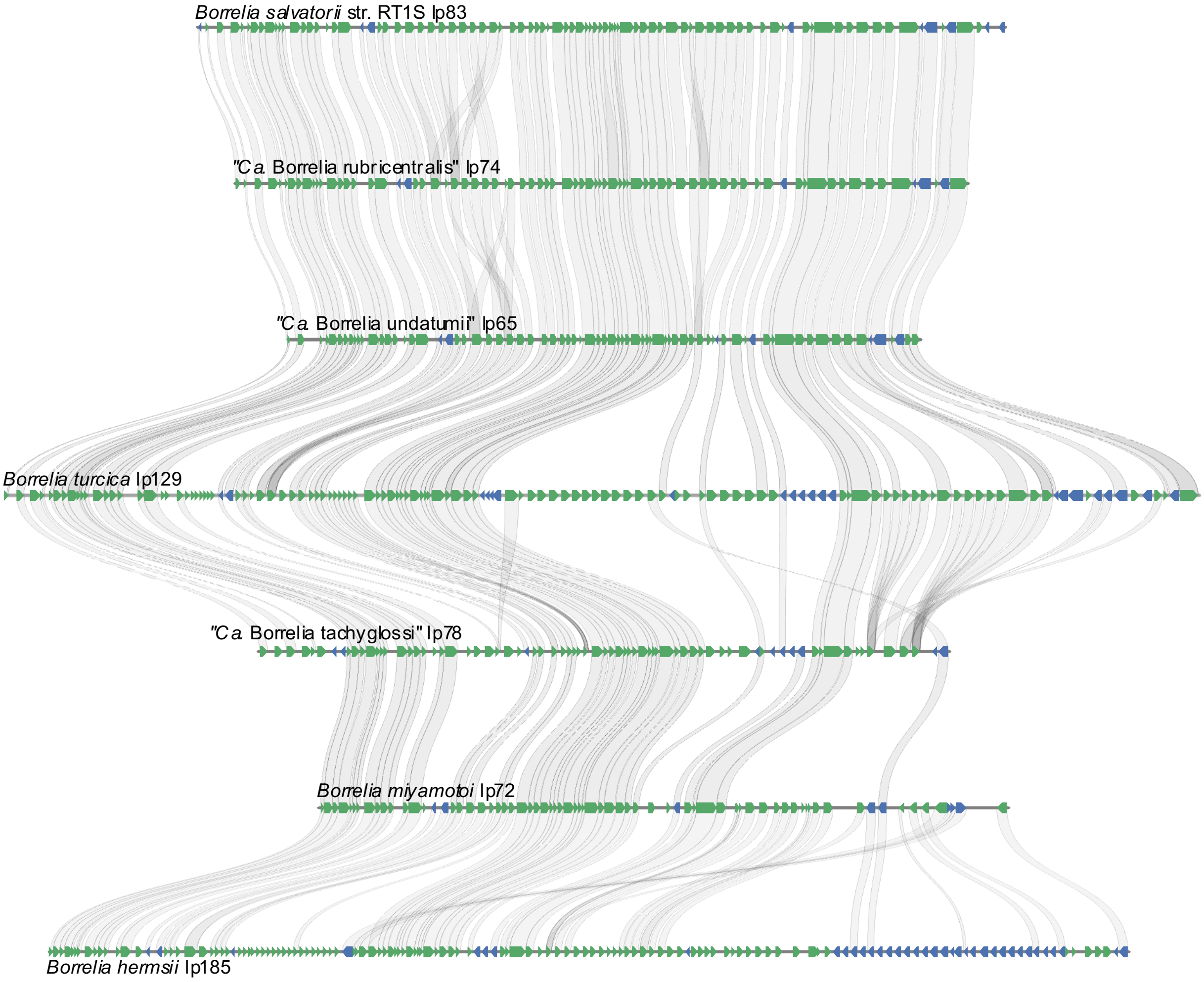

Figure S3. MCscan plot showing orthologous protein coding genes between relapsing fever and reptile-associated *Borrelia* linear 'megaplasmids'.

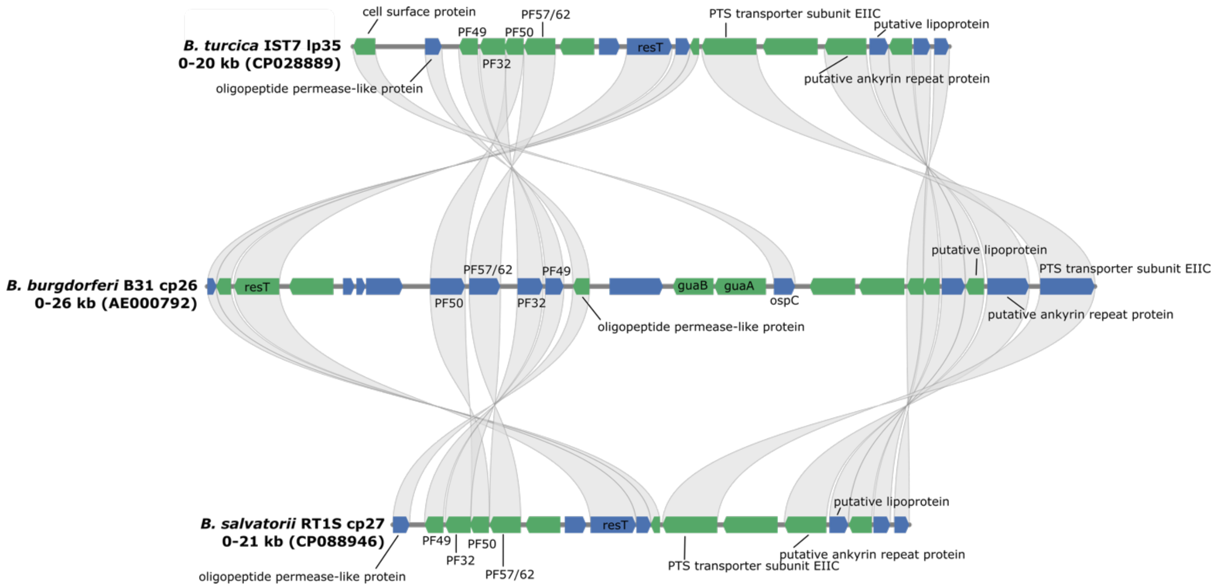

Figure S4. MCscan plot showing orthologous proteins coding genes between *B. burgdorferi* B31 plasmid cp26 and *B. turcica* IST7 plasmid lp25 and *B. salvatorii* RT1S cp27.

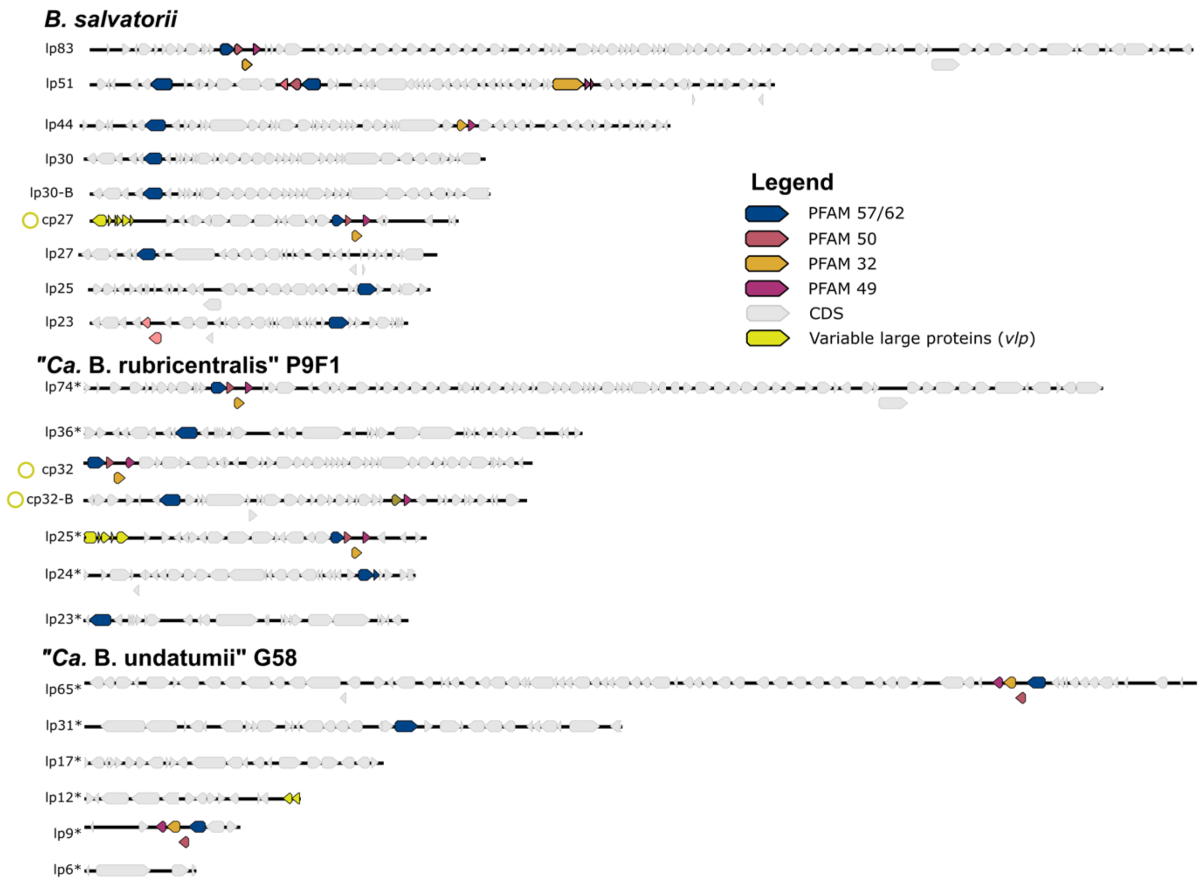

Figure S5 Schematic of Varanid-associated *Borrelia* spp. and the approximate locations of putative plasmid partitioning genes, and variable large proteins.

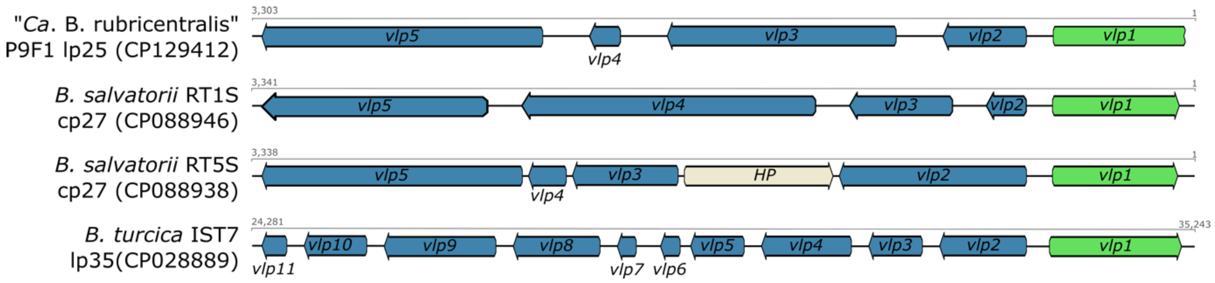

Figure S6 Schematic gene map of *vlp* gene clusters in REP *Borrelia* replicons, including *vlp1* (green) which was homologous in all genomes. HP: hypothetical protein.

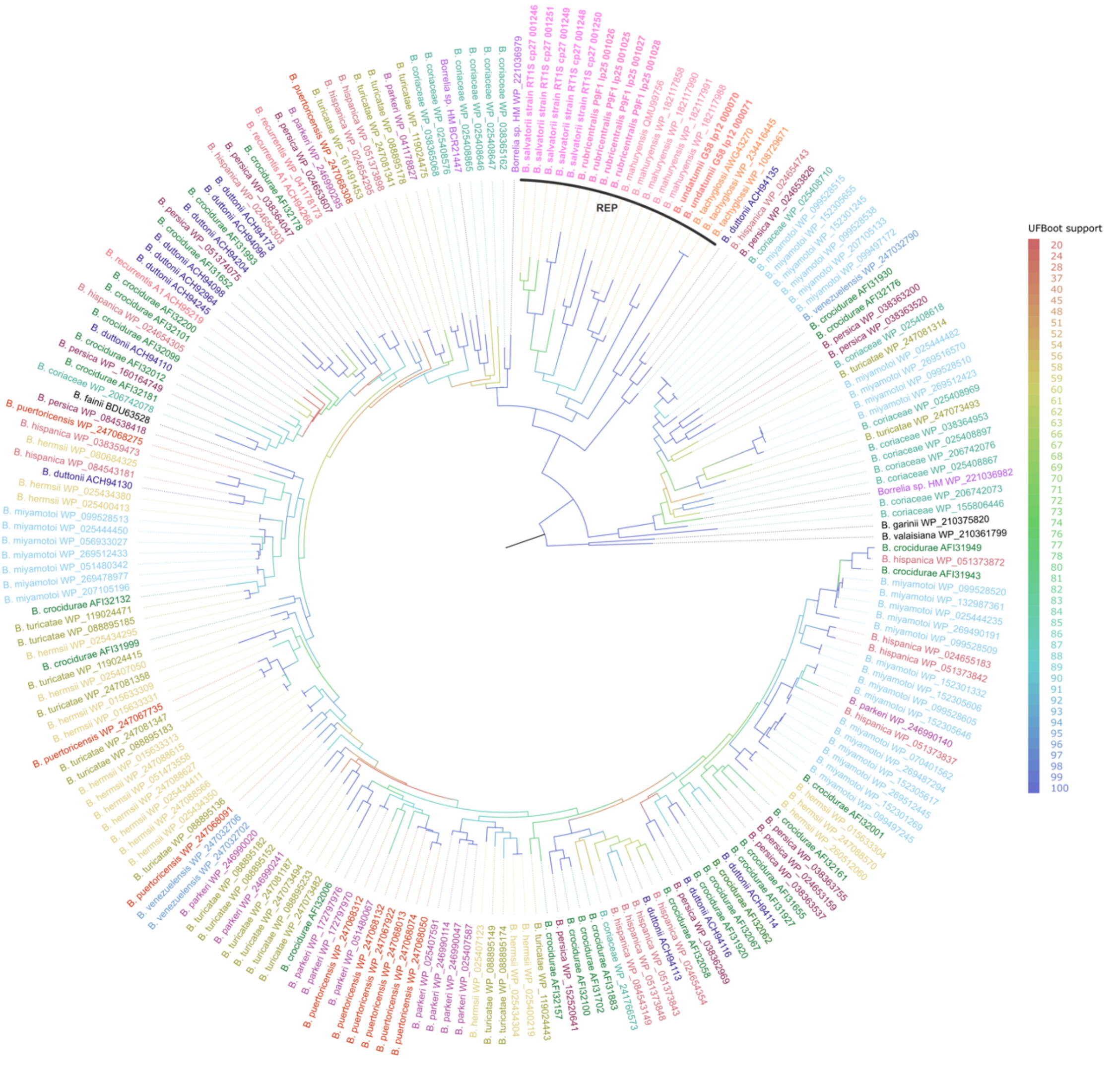

Figure S7 Phylogenetic reconstruction of REP and RF *Borrelia vlp* amino acid sequences. Branches are colour coded according to bootstrap support and *Borrelia* species are individually colour coded. REP *Borrelia vlp* sequences are highlighted by the outer ring. Tree constructed with IQ-TREE v.2.2.0 with model selection and 1000 ultrafast bootstrap replicates.

Extended caption for Fig. 5

The isolate, ORF#, and plasmid is given for each sequence. isolate prefixes: Bsal-RT1S, *B. salvatorii* RT1S; Bsal-RT5S, *B. salvatortii* RT5S; Brub, *B. rubricentralis;* Bund, *B. undatum*; Btur, *B. turcica* IST7; BmahAFGy1, *B. mahuryensis* AFGy1; BtachBcF101268, *B. tachyglossi* BcF101268; baBA2, *B. anserina* BA2; bhDAH, *B. hermsii* DAH; bhYOR, *B. hermsii* YOR; bcCo53, *B. coriaceae* Co53; bpuSUM, *B. puertoricensis* n. sp. SUM; bpSLO, *B. parkeri* SLO; bvRMA01, *B. venezuelensis* RMA01; bt91E135, *B. turicatae* 91E135; btBTE5EL, *B. turicatae* BTE5EL; DB299, *B. bavarensis* Pbi; BBIDN127, *B. bissettii* DN12; BB, *B. burgdorferi* B31; BBUJD1, *B. burgdorferi* JD1; Bmayo, *B. mayonii* MN14-1420; AXH25, *B. miyamotoi* CT13-2396. Reference PF32 and PF57/62 sequences were sourced from [31].
